# Single-source chest-abdomen-pelvis cancer staging on a third generation dual-source CT system: comparison of automated tube potential selection to second generation dual-source CT

**DOI:** 10.1186/s40644-016-0093-1

**Published:** 2016-10-10

**Authors:** Clara Park, Tatjana Gruber-Rouh, Doris Leithner, Amelie Zierden, Mortiz H. Albrecht, Julian L. Wichmann, Boris Bodelle, Mohamed Elsabaie, Jan-Erik Scholtz, Moritz Kaup, Thomas J. Vogl, Martin Beeres

**Affiliations:** Department of Diagnostic and Interventional Radiology, Clinic of the Goethe University, Haus 23C UG, Theodor-Stern-Kai 7, 60590 Frankfurt, Germany

**Keywords:** Multidetector Computed Tomography, Cancer, Cancer Staging, Neoplasms, Automated Tube Potential Selection, Dual-Source CT

## Abstract

**Background:**

Evaluation of latest generation automated attenuation-based tube potential selection (ATPS) impact on image quality and radiation dose in contrast-enhanced chest-abdomen-pelvis computed tomography examinations for gynaecologic cancer staging.

**Methods:**

This IRB approved single-centre, observer-blinded retrospective study with a waiver for informed consent included a total of 100 patients with contrast-enhanced chest-abdomen-pelvis CT for gynaecologic cancer staging. All patients were examined with activated ATPS for adaption of tube voltage to body habitus. 50 patients were scanned on a third-generation dual-source CT (DSCT), and another 50 patients on a second-generation DSCT. Predefined image quality setting remained stable between both groups at 120 kV and a current of 210 Reference mAs.

Subjective image quality assessment was performed by two blinded readers independently. Attenuation and image noise were measured in several anatomic structures. Signal-to-noise ratio (SNR) was calculated. For the evaluation of radiation exposure, CT dose index (CTDI_vol_) values were compared.

**Results:**

Diagnostic image quality was obtained in all patients. The median CTDI_vol_ (6.1 mGy, range 3.9–22 mGy) was 40 % lower when using the algorithm compared with the previous ATCM protocol (median 10.2 mGy · cm, range 5.8–22.8 mGy). A reduction in potential to 90 kV occurred in 19 cases, a reduction to 100 kV in 23 patients and a reduction to 110 kV in 3 patients of our experimental cohort. These patients received significantly lower radiation exposure compared to the former used protocol.

**Conclusion:**

Latest generation automated ATPS on third-generation DSCT provides good diagnostic image quality in chest-abdomen-pelvis CT while average radiation dose is reduced by 40 % compared to former ATPS protocol on second-generation DSCT.

## Background

Contrast-enhanced chest-abdomen-pelvis computed tomography (CT) is the standard imaging procedure for oncologic staging. It is widely available and allows evaluation of several anatomic structures such as lymph nodes, abdominal organs, lung and bony structures to detect malignancies in one comprehensive examination. Due to the increased amount of overall CT examinations [1, 2], radiation exposure should be used with care following the ‘as low as reasonably achievable’ (ALARA) rule. Nevertheless, cancer might be life-limiting and cancer staging overweight risks of radiation exposure [3–6]. However, patients with a detected malignancy in an early stage might receive multiple follow-up CT scans with an increased cumulative radiation dose and, therefore, an increased risk of radiation dose related disease [4].

Many techniques have been introduced to lower radiation exposure such as automated attenuation-based tube current and voltage modulation, noise reduction filters, and iterative reconstruction algorithms [2, 6–15]. Automated attenuation based tube potential selection (ATPS) is routinely used in our department for oncologic staging and follow-up chest-abdomen-pelvis CT [16, 17].

The aim of our study is to evaluate image quality and radiation exposure of latest generation automated tube potential selection on a third-generation dual-source CT (DSCT) in comparison to the second-generation DSCT in gynaecologic oncology staging and follow up.

## Methods

### Patients

This retrospective study was performed as a single-centre, observer-blinded study. The institutional review board approved this study; written informed consent requirement was waived. A total of 100 patients (100 women, median age 62.5 [range 31–86 years]) underwent contrast-enhanced chest-abdomen-pelvis CT examinations for gynaecologic oncology staging between October 2013 and March 2016. General exclusion criteria for contrast-enhanced CT included impaired renal function (estimated glomerular filtration rate <60 mL/min, calculated by creatinine blood level and patient age), hyperthyroidism, as well as hypersensitivity to iodine contrast media. Non-contrast scans were excluded.

In Group 1 50 patients were examined on a second-generation 128-slice DSCT (Somatom Definition Flash, Siemens Healthcare, Forchheim, Germany) using former generation of ATPS (CAREkV, Siemens, Forchheim, Germany) with steps of 20 kV between 80 kV and 140 kV. Group 2 included 50 patients who were examined on a third-generation 192-slice DSCT (SOMATOM Force, Siemens Healthcare) with the latest generation of ATPS which allows tube potential selection in steps of 10 kV between 70 kV and 150 kV. Both imaging protocols were adjusted to a pre-defined image quality of 120 kV/210 Ref.mAs (Table 1). Patient populations were paired regarding age, body size and habitus.

**Table 1:** Study population and evaluation of examination parameters

	Group 1	Group 2
Imaging mode	Single-source	Single-source
Machine	Definition Flash	Definition Force
Slice x collimation	128 × 0.6	192 × 0.6
Pitch	1.2	1.2
kV/ref. mAs	120/210 (CarekV)	120/210 (CareDose4D)
Patients 90 kV		19
Patients 100 kV	30	23
Patients 110 kV		3
Patients 120 kV	16	4
Patients 130 kV		
Patients 140 kV	4	
Patients 150 kV		1

### Automated attenuation-based kV selection

ATPS selects the optimal tube potential based on the selected body region, the scout and a 12-point scale which allows manual selection of desired examination type to optimize for CT-angiography, parenchymal contrast-enhanced CT or visualization of bony structures. [17]. This is useful, because for a high-contrast situation used in CT-angiography, lowering of the kV leads to higher absorption and higher attenuation values of the vascular structures with additional possibility to safe radiation dose in low-kV CT scans. In this study we set focus to an optimal contrast-enhanced parenchymal organ scan for cancer staging.

The automatically selected kV remained stable throughout the CT scan. In addition, a real-time automatic mAs-modulation software (CARE Dose 4D, Siemens Healthcare) was used to further reduce radiation exposure.

### CT examination

Two hours before the actual CT examination patients were told to start drinking oral contrast media (Micropaque, Guerbet, Villepinte, France) for improved gastrointestinal delineation. Before the start of the CT scan, an intravenous contrast material bolus, containing 1 ml/kg of iodinated contrast material (Ultravist 370, Bayer-Schering, Germany), followed by a saline chaser of 40 mL was injected at 2 mL/s flow rate via an antecubital vein using a double-syringe power injector (CT2, Medtron, Saarbruecken, Germany).

A fixed delay of 70 s post injection was used in order to obtain venous contrast. All CT examinations were performed in a cranio-caudal direction starting from the upper thorax aperture down to the femoral ligaments. In group 1 at a collimation of 128 · 0.6 mm, pitch 1.2, and rotation time of 0.5 s, in group 2 at a collimation of 192 · 0.6 mm, pitch 1.2, and rotation time of 0.5 s.

### CT data reconstruction

For fast overviewing, images were reconstructed in 5-mm slice thickness with a 5-mm increment. For detailed evaluation, data were additionally reconstructed with a slice thickness of 3 mm and increment of 1.5 mm using a medium-smooth soft-tissue convolution kernel (B30f) for parenchymal analysis and a hard convolution kernel for the analysis of bony structures and the lungs (B70f). To allow an image comparison independently of different available iterative reconstruction algorithms (Safire on 2^nd^ generation DSCT, ADMIRE on 3^rd^ generation DSCT, Siemens Healthcare), we selected filtered-back projection (FBP) reconstruction algorithms by purpose for both groups.

### Radiation dose estimations

CTDI_vol_ and DLP values displayed in the patient’s protocol were recorded.

### Image quality

Subjective image quality was assessed by two radiologists individually with 4 and 7 years of experience in whole body imaging on a five-point rating scale : 1 = excellent: excellent definition of tumour and/or metastases, excellent delineation of the structures; 2 = good: good definition of tumour and/or metastases, minimal image noise; 3 = adequate: adequate definition of tumour and/or metastases, slight impact of image noise, sufficient for diagnosis; 4 = poor: poor definition of tumour and/or metastases, low attenuation and difficult delineation of the structures, increased image noise, diagnostic confidence reduced; 5 = unacceptable/nondiagnostic. The most probable reasons for reduced image quality such as obesity, motion, metallic artefacts, contrast medium flow-related, and contrast timing were noted.

Objective image quality analysis was performed by one radiologist with 7 years of experience in general radiology on a regular PACS workstation (Centricity 4.2, General Electric Healthcare, Munich, Germany). Regions of interest (ROI) were drawn in several anatomic regions such as aorta at the level of the pulmonary trunk, both lobes of the lung, left lobe of the liver, right lobe of the liver, pancreas, spleen, kidneys, gluteus maximus muscle, pelvic bone and pre-sternal in the air at the level of the pulmonary trunk. Attenuation and standard deviation of each ROI were noted. Each measurement was performed four times to minimize bias. Average values for attenuation and standard deviation (SD) of each region were calculated. Background noise was determined as the standard deviation (SD) of the ROI in the air. Signal-to-noise ratio (SNR) was determined according to the following equation: SNR = attenuation/background noise (Table 3).

Tumour and/or metastases were evaluated and measured using tumour diameters for objective evaluation and, for subjective scoring, we evaluated whether the imaging modality might have affected the tumour staging.

### Statistical analysis

Statistical analysis was performed using dedicated software (Stata/IC 13, Stata Corp, College Station, Texas, USA). Continuous variables were reported as median and range, categorical variables as frequencies or percentages.

Radiation parameters and quantitative image parameters (e.g. noise, attenuation) were tested using the Wilcoxon Mann–Whitney *U* test as the data were nonparametric. The relationship between patient diameter and automated kV selection was analysed using the Spearman rank order correlation test. The Chi-square (*X*2) test was used for categorical variables (demographic patient data). Statistical significance was defined as a p-value above 0.05. Differences between the both readers were assessed using Cohen’s kappa (j) interpreted in the following way: j\0.20, slight agreement; j = 0.21–0.40, fair agreement; j = 0.41–0.60, moderate agreement; j = 0.61–0.80, substantial agreement; j = 0.81–1.0, almost perfect agreement.

## Results

### Radiation dose estimation

**Fig. 1:**
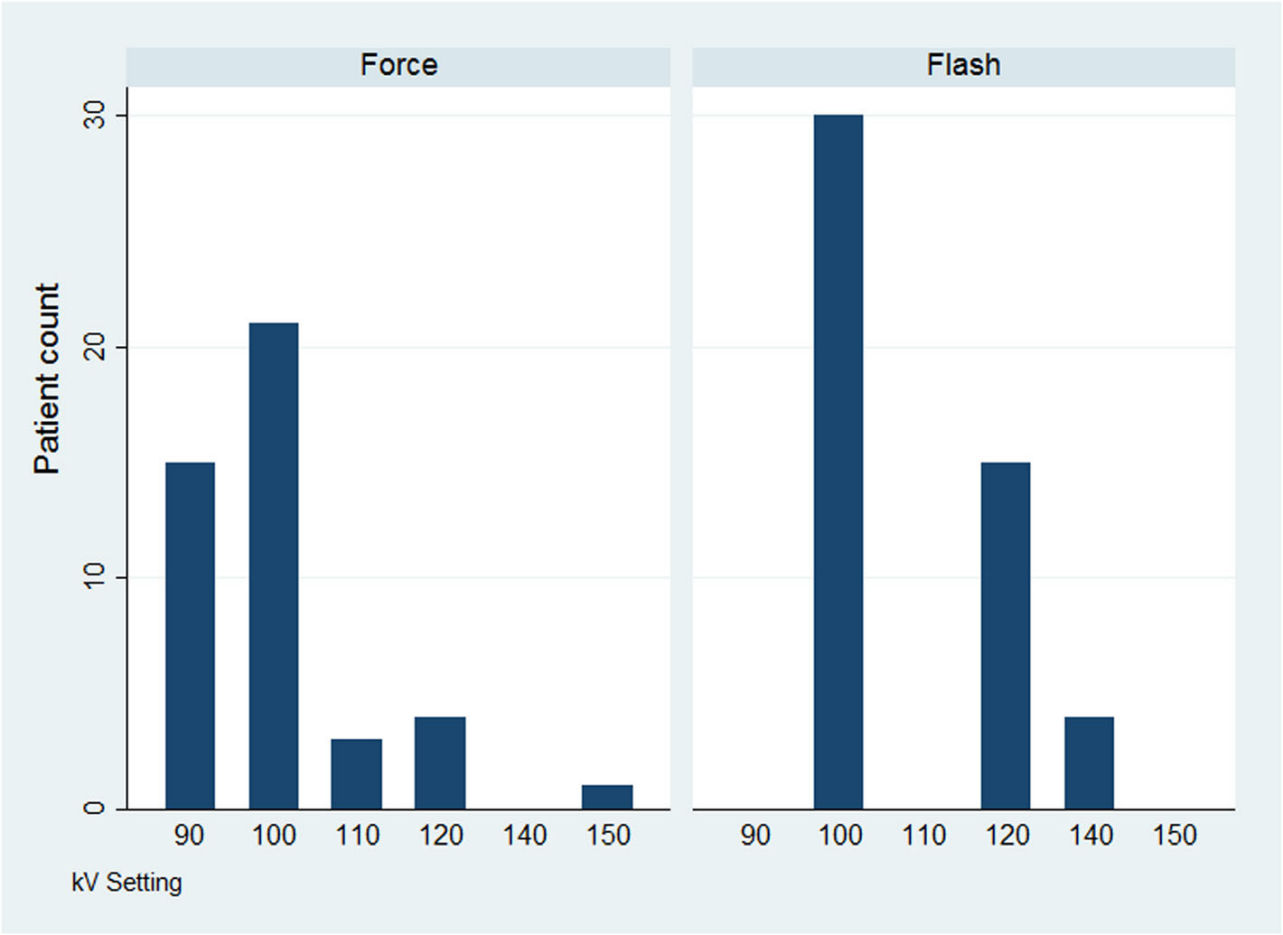
Choosen kV between both groups

In group 1 ATPS resulted in a tube voltage selection of 100 kV in 30 patients, 120 kV in 16 patients and 140 kV in 4 patients. ATPS resulted in a tube voltage reduction to 90 kV in 19 cases, 100 kV in 23 patients, and 110 kV in 3 patients in group 2. 4 Patients were examined at 120 kV and 1 patient at 150 kV (Fig. 1, Table 1).

The median CTDIvol in group 2 was 4.8 mGy (3.9–9.8 mGy) at 90 kV, 6.3 mGy (5.3–9.0 mGy) at 100 kV, 9.7 mGy (6.6–14.1 mGy) at 110 kV, 11.5 (7.8–14.4 mGy) at 120 kV and 22.0 mGy (only one patient) at 150 kV. In the baseline group the median CTDIvol was 8.9 mGy (5.8–14.6 mGy) at 100 kV, 13.2 mGy (7.1–17.4 mGy) at 120 kV and 20.9 mGy (20.1–22.8 mGy) at 140 kV.

The overall comparison between both groups revealed a CTDIvol of 6.1 mGy (3.9–22.0 mGy) in the group 2 and a CTDIvol of 10.2 mGy (5.9–22.8 mGy) in group 1. Comparing both groups revealed a statistical significant difference with a p-value < 0.01 (Table 2).

**Table 2:** Examination parameters

	Group 1	Group 2	*p*-Value: Group 1 vs. Group 2
Patients (all female)	50	50	
Age (years)			
Scanning range (cm)	63.9 (42.4–75.3)	66.1 (41.3–78.1)	0.2
CTDI	10.2 (5.8–22.8)	6.1 (3.9–22.0)	0.01
Dose-length product (mGy x cm)	684 (420–1399)	376.2 (209.1–1406.7)	0.01

Median lateral patient diameter was 36.8 cm (30.7–47.4 cm) in group 1 in comparison to 37.2 cm (32.2–47.5 cm) in group 2 (*p* = 0.2, Tables 3 and 4). Transversal patient diameter was similar in group 1 (mean, 21.3 cm; range, 12.2–33.0 cm) compared to group 2 (mean, 21.8 cm; range 17.3–33.1 cm;*p* = 0.4) (Table 3).

**Table 3:** Study population – detailed overview

	Group 1	Group 2	*p*-Value: Group 1 vs. Group 2
Image noise	7.4 (4.4–40.8)	7.9 (3.5–23.1)	0.8
SNR	25.2 (4.6–38.5)	27.1 (4.3–49.5)	0.08
Patient diameter lateral	36.8 (30.7–47.4)	37.2 (32.2–47.5)	0.2
Patient diameter transversal	21.3 (12.2–33.0)	21.8 (17.3–33.1)	0.4

**Table 4:** Correlation Analysis – Body Diameter/Radiation Dose (the asterisk* marked part should be evaluated with caution because of low patient count)

		Patients	CTDIvol (mGy)	Total mAs	Body Diam. sag	Body diam. Transv.	Spearman correlation coefficient (rho) – lateral diameter/CTDIvol	Spearman correlation coefficient (rho) – transverse diameter/CTDIvol
Group 1	Patients 100 kV	30	8.9 (5.8–14.6)	217 (179–355)	32.5 (27.1–41.2)	21.2 (14.9–30.4)	0.80	0.89
	Patients 120 kV	16	10.8 (7.1–17.4)	213 (152–257)	34.1 (27.9–45.8)	24.4 (16.7–35.1)	0.81	0.83
	Patients 140 kV	4	20.9 (20.1–22.8)	218 (204–232)	43.8 (39.8–48.4)	29.5 (24.7–31.5)	0.65*	0.32*
Group 2	Patients 90 kV	19	4.8 (3.9–9.8)	165.0 (134.0–338.0)	34.5 (31.9–39.0)	20.4 (18.2–24.3)	0.71	0.43
	Patients 100 kV	23	6.3 (5.3–9.0)	156.0 (130.0–224.0)	38.0 (32.3–44.1)	21.6 (12.2–27.3)	0.68	0.71
	Patients 110 kV	3	9.7 (6.6–14.1)	185.0 (125.0–270.0)	40.4 (30.7–47.4)	22.7 (17.6–33.0)	1.0 *	1.0 *
	Patients 120 kV	4	11.5 (7.8–14.4)	171.0 (106.0–189.0)	37.8 (33.4–41.4)	23.1 (18.9–28.0)	0.2 *	1.0 *
	Patients 150 kV	1	22.01	192.0	42.2	28.7	N/A	N/A

### Image quality

Diagnostic image quality was obtained from all patients in group 2 (excellent: *n* = 47; good: *n* = 2; moderate: *n* = 1). The reasons for moderate image quality were discussed between both readers and, in all cases, due to difficulties in ruling out parenchymal lesions, some because of image noise, and some because of insufficiencies concerning the venous contrast enhancement.

Concerning image quality in the control group, it was also rated as sufficient in all cases (excellent: *n* = 44 good: *n* = 5; moderate: *n* = 1).

Inter-reader agreement on measurements was almost perfect (j = 0.82).

**Fig. 2:**
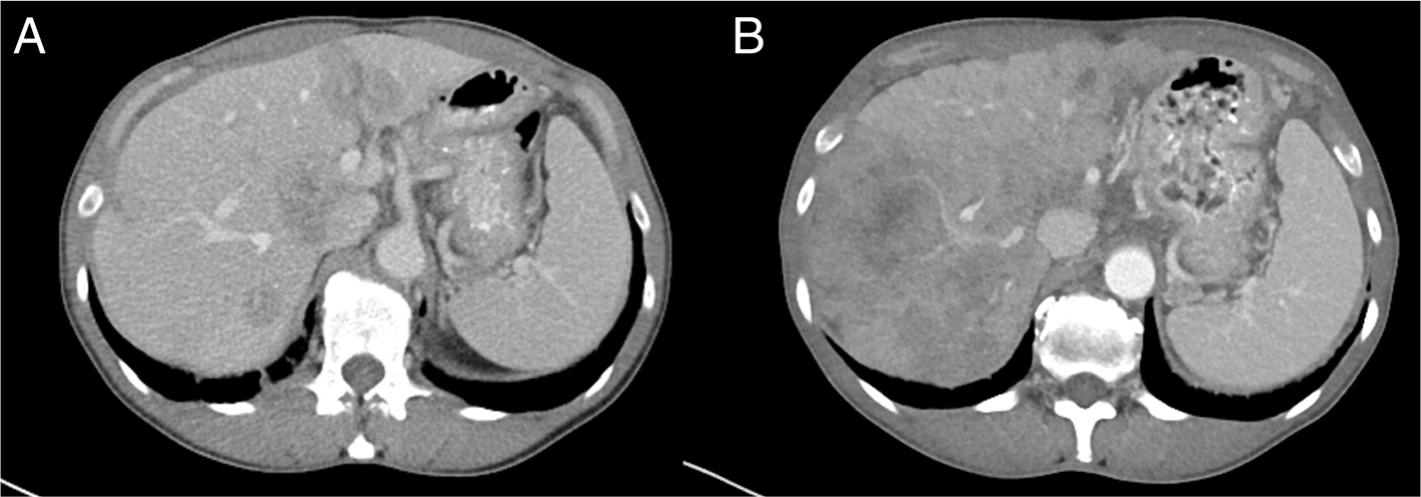
Image quality comparison in parenchymal liver lesions. Same patient in progressive disease. **a** Flash-CT: CTDIvol 14.40 mGy, DLP 1035 mGy · cm. **b** Force-CT: CTDIvol 13.35 mGy, DLP 946 mGy · cm

**Fig. 3:**
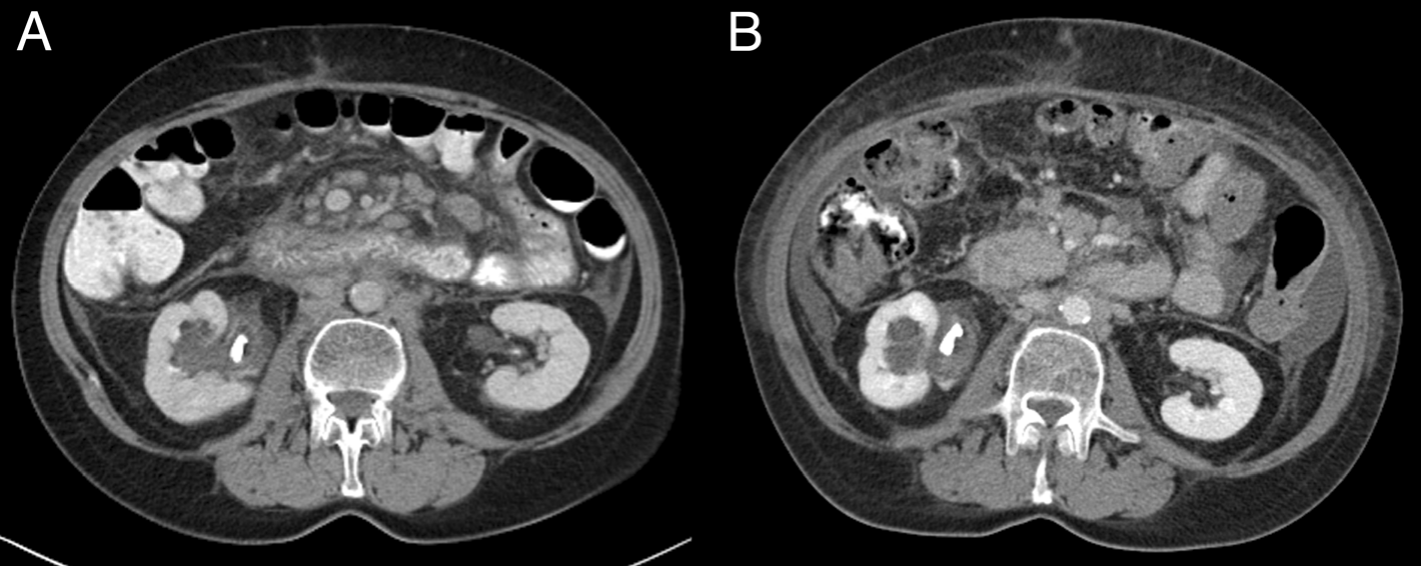
Image quality comparison in para-aortic lymph-nodes. Same patient in progressive disease. **a** Flash-CT: CTDIvol 7.72 mGy, DLP 516 mGy · cm. **b** Force-CT: CTDIvol 3.88 mGy, DLP 278.5 mGy · cm

In summary, the image quality rating demonstrated, that all malignancies could be ruled out adequately and classified in a correct manner with respect to RECIST (Response Evaluation Criteria In Solid Tumours), without statistically significant differences between the two groups (*p* = 0.4, Figs. 2 and 3) [18].

## Discussion

Our results showed a reduction in the median CTDI_vol_ of 40 % when using latest generation ATPS compared with the previous ATPS protocol. One of the first studies on ATCM performed by Winklehner et al. [19] evaluated automated attenuation-based kV selection in 40 patients for CT angiography of the aorta. In this study, an overall radiation dose reduction of 25.1 % was observed, while keeping the image quality stable, when using a 120 kV protocol.

Eller et al. [20] evaluated automated attenuation-based kV selection in 100 patients. They carried out an abdominal CT examination for 52 of the patients, and a thoraco-abdominal examination for 48 patients. All examinations using automated attenuation-based kV selection resulted in a radiation dose reduction of at least 11.4 %; in detail, 13.2 % in the abdominal CT group and 9.5 % in the thoraco-abdominal group.

Gnannt et al. [21] assessed automatic attenuation-based kV selection in 40 patients suffering from testicular cancer. In this study, a CT scan of the chest was performed in a mixed arterio-venous phase and the abdominal CT examination was carried out in the portal-venous phase of enhancement. The overall dose reduction was 12 % on average.

With focus on cancer staging, Beeres et al. evaluated automated tube potential selection in 110 patients compared to a 120 kV automated tube potential selection protocol [17]. The overall dose reduction was 7.9 % in a randomly chosen cohort.

In all the studies mentioned above, there was no statistically significant worsening of subjective image quality. In the study by Beeres et al. [17], there was a dose reduction of 7.9 %, this is comparable to the study of Eller et al. [20] where there was a dose reduction of 9.5 % in chest-abdomen-pelvis CT examinations reported.

A recently published study of Scholtz et al. compared ATPS on a second-generation DSCT with FBP and third-generation DSCT in combination with a new advanced modelled iterative reconstruction algorithm resulting in an average overall dose reduction of 34.9 % [16]. However, in our study we used FBP in both groups to compare the plain dataset and to prevent from influence of iterative reconstruction algorithm.

Our study showed an overall dose reduction in 40 % of the study population which meets with the study above. The main driver in this dose reduction setting might be the possibility of the tube to select the potential in 10 kV steps compared to the former software. The tube potential in our study switched only once to 150 kV compared with the cited studies, and a tube potential of 80 kV or below wasn’t automatically chosen in any case (Table 1).

In vascular imaging, it is possible to examine the region of interest using a lower kV setting because of the high-contrast situation attained by the arterial phase of the contrast material. In contrast, when parenchymal contrast is the clinical setting and the object in question, for example, when liver lesions have to be ruled out, automated attenuation-based tube potential selection might not lower the kV in the same aggressive manner as in vascular imaging (Fig. 1).

Applying the new algorithm combined with an x-ray tube system that is able to switch between different kV levels in 10 kV steps, an overall radiation dose reduction of 40 % was possible.

### Limitations

Some limitations of our study need to be addressed. First, the overall number of patients in our study was small; further studies with a larger cohort are required.

Second, we did not record the body mass index, but measured patient diameter as an alternative. Third, while a patient cohort of 100 patients is sufficient for an initial experience, additional larger studies are necessary to assess further potential of this technique.

Fourth, we did not investigate iterative reconstruction algorithms and the effects of ATCM and iterative reconstructions in our study. However, this has been evaluated in prior studies [16, 22].

## Conclusion

Our results demonstrate that ATPS in third-generation DSCT allows tube voltage selection in steps of 10 kV resulting in an average dose reduction of 40 % compared to second-generation DSCT and is feasible for oncologic chest-abdomen-pelvis CT examination in clinical routine while overall image quality remains excellent.

## Abbreviations

ALARA: As low as reasonably achievable
ATCM: Automated tube current modulation
ATPS: Automated attenuation-based tube potential selection
CT: Computed tomography
CTDIvol: CT dose index
DLP: Dose-length-product
kV: Kilovolt
mAs: Miliampere-second
SNR: Signal-to-noise ratio
